# Differential remodeling of subcutaneous white and interscapular brown adipose tissue by long-term exercise training in aged obese female mice

**DOI:** 10.1007/s13105-023-00964-2

**Published:** 2023-05-19

**Authors:** Elisa Félix-Soriano, Neira Sáinz, Eva Gil-Iturbe, Rosa Castilla-Madrigal, Jon Celay, Marta Fernández-Galilea, Álvaro Pejenaute, M. Pilar Lostao, José A. Martínez-Climent, María J. Moreno-Aliaga

**Affiliations:** 1 University of Navarra; Center for Nutrition Research and Department of Nutrition, Food Science and Physiology; School of Pharmacy and Nutrition, Pamplona, Spain; 2 Division of Hemato-Oncology, Center for Applied Medical Research CIMA, University of Navarra, Pamplona, Spain; 3 CIBERONC, Instituto de Salud Carlos III (ISCIII), Madrid, Spain; 4 CIBER de Fisiopatología de la Obesidad y Nutrición (CIBEROBN). Instituto de Salud Carlos III (ISCIII), Madrid, Spain; 5 IdiSNA, Navarra Institute for Health Research, Pamplona, Spain

**Keywords:** Aging, Obesity, Treadmill training, White adipose tissue, Brown adipose tissue, Female

## Abstract

Obesity exacerbates aging-induced adipose tissue dysfunction. This study aimed to investigate the effects of long-term exercise on inguinal white adipose tissue (iWAT) and interscapular brown adipose tissue (iBAT) of aged obese mice. Two-month-old female mice received a high-fat diet for 4 months. Then, six-month-old diet-induced obese animals were allocated to sedentarism (DIO) or to a long-term treadmill training (DIOEX) up to 18 months of age. In exercised mice, iWAT depot revealed more adaptability, with an increase in the expression of fatty acid oxidation genes (*Cpt1a*, *Acox1*), and an amelioration of the inflammatory status, with a favorable modulation of pro/antiinflammatory genes and lower macrophage infiltration. Additionally, iWAT of trained animals showed an increment in the expression of mitochondrial biogenesis (*Pgc1a, Tfam, Nrf1*), thermogenesis (*Ucp1*), and beige adipocytes genes (*Cd137, Tbx1*). In contrast, iBAT of aged obese mice was less responsive to exercise. Indeed, although an increase in functional brown adipocytes genes and proteins (*Pgc1a*, *Prdm16* and UCP1) was observed, few changes were found on inflammation-related and fatty acid metabolism genes. The remodeling of iWAT and iBAT depots occurred along with an improvement in the HOMA index for insulin resistance and in glucose tolerance. In conclusion, long-term exercise effectively prevented the loss of iWAT and iBAT thermogenic properties during aging and obesity. In iWAT, the long-term exercise program also reduced the inflammatory status and stimulated a fat-oxidative gene profile. These exercise-induced adipose tissue adaptations could contribute to the beneficial effects on glucose homeostasis in aged obese mice.

**Supplementary Information:**

The online version contains supplementary material available at 10.1007/s13105-023-00964-2.

## Introduction

In the past years, the existence of three major types of adipose tissue, white, brown and beige, has been evidenced [59]. The 95% of the adipose mass is constituted by white adipose tissue (WAT), which main function is energy storage as triglycerides [59]. However, WAT also participates in whole-body metabolic regulation through the production of adipokines [59]. WAT is organized into visceral and subcutaneous depots, which are heterogeneous and show marked metabolic differences. Therefore, while visceral WAT accumulation is associated to insulin resistance and whole-body metabolic disturbances, the accumulation of triglycerides in subcutaneous depots does not seem to have deleterious effects and could be beneficial to prevent metabolic syndrome [38].

On the other hand, brown adipose tissue (BAT) is a thermogenic tissue that dissipates energy as heat mainly through the uncoupling protein 1 (UCP1). Although BAT represents only 1–2% of body fat [38], BAT is relevant for the regulation of energy homeostasis and the prevention of obesity [14]. Moreover, BAT activity has also been involved in the regulation of glucose homeostasis and triglycerides clearance [38, 56]. Finally, the third type of adipose tissue is constituted by beige adipocytes, which display an intermediate phenotype between white and brown adipocytes, but constitute a distinct type of thermogenic fat cell [92] found mainly in the subcutaneous WAT [5]. Importantly, the appearance of these thermogenic beige adipocytes in white adipose depots can be induced by several stimuli through a process termed beiging of WAT [5]. Hence, activation of classical brown adipocytes, as well as the induction of beige adipocytes, have been proposed as anti-obesity and associated metabolic disorders targets [101].

During aging, fat distribution shifts from subcutaneous to visceral fat depots, while both BAT activity and WAT beiging are decreased [6, 101]. These alterations can be accelerated by obesity [47, 61]. On the other hand, the dysfunctional WAT and BAT that develop during aging and obesity have been associated to the local chronic, low-grade inflammation that accompanies both processes [71–73, 88].

In this context, physical exercise has been recognized to be a therapeutic and preventive approach for cardiovascular disease, diabetes, obesity, certain types of cancer, functional and cognitive decline, dementia, and indeed, for a healthy aging [1, 12, 45, 62, 76, 84, 95]. These beneficial actions are mediated by the exercise-induced modulation of physiological functions in many organs [32]. Certainly, physical exercise is able to prevent age-related disorders, including visceral fat accumulation, skeletal muscle loss and age-related inflammation in animal experimental models [58]. In young adult mice, short-term exercise (*i.e.* 3 weeks) is sufficient to directly improve the metabolism of the different adipose depots [46]. Specifically in WAT, exercise improves glucose uptake and insulin sensitivity, induces smaller adipocytes, regulates lipolysis, increases mitochondrial activity, reduces inflammation, promotes beiging, modulates adipokine secretion and slows down cellular senescence [28, 41, 42, 74, 81, 82, 91] The effects on BAT, however, are more controversial. Indeed, exercise has been shown to induce BAT thermogenic activation, but also no effects, or even an exercise-induced inhibition, have been observed [16, 19, 46, 93]. On the other hand, the effects on BAT glucose and fatty acid metabolism, as well as on mitochondrial biogenesis, suggest that there are no adaptations to BAT metabolism [reviewed in 86].

Importantly, the adipose depots also contribute to the exercise-induced metabolic effects. Indeed, subcutaneous WAT plays a key role in the exercise-induced improvements in whole-body glucose homeostasis. Hence, the transplantation of subcutaneous WAT from exercise-trained mice to high-fat diet (HFD)-induced obese (DIO) mice reversed the deleterious effects of high-fat feeding on glucose tolerance and insulin sensitivity [81]. Although the metabolic contributions of BAT are not as studied, it has been shown that exercise induces the release of BAT adipokines (*batokines*) that act to improve the functions of other metabolic organs, such as muscle fatty acid uptake [80].

Despite the growing evidence revealing beneficial effects of exercise training on WAT and BAT metabolism and inflammation, these studies have been conducted mainly in young adult but not in older animal models of obesity. Among these studies, 8 weeks of exercise were proven to improve visceral WAT and the associated disturbances in lipid metabolism biomarkers in old DIO mice, together with an increase in WAT fatty acid oxidation [2]. Moreover, few studies have analyzed the beneficial effects of long-term exercise started at midlife or aging [54, 58]. In this regard, the actions of exercise on aging per se included a lower body weight [58] and a preventive effect on the appearance of the metabolic and energy expenditure disturbances associated to aging [54]. Interestingly, this preventive effect was more pronounced in female animals [54].

Therefore, we aimed to characterize if a long-term exercise program started at the late adulthood could prevent the alterations induced by aging and diet-induced obesity on glucose homeostasis, as well as on WAT and BAT lipid metabolism and inflammatory status, together with the maintenance/stimulation of markers of BAT activity and WAT browning in aged, obese female mice.

## Materials and Methods

### Animal experimental design

Female C57BL6/J mice were purchased at 7 weeks of age from Harlan Laboratory (Barcelona, Spain) and housed at the animal facilities of the University of Navarra under controlled conditions (22 ± 2 °C, 12-h light/12-h dark cycle; relative humidity 55% ± 10%). After 10 days of acclimation, animals started consuming a high-fat diet (HFD) providing 20% kcal as proteins, 35% as carbohydrates, and 45% as lipids (Research Diets Inc., New Brunswick, N.J., USA) for 4 months to induce obesity. Then, 6-month-old DIO animals were maintained on the HFD and divided into two experimental groups: a DIO sedentary group (DIO, n = 10) and a DIO + exercise group (DIOEX, n = 8), which performed a treadmill running protocol up to 18 months of age.

All experimental groups were fed ad libitum and controlled for body weight 3 days/week during the whole experiment. Before sacrifice, animals underwent an in vivo body composition determination and a glucose tolerance test (GTT). At the end of the experiment, overnight-fasted animals were sacrificed and fat depots, including subcutaneous-inguinal (iWAT), visceral WAT (mesenteric, gonadal, retroperitoneal), and interscapular BAT (iBAT) were collected, weighted and frozen at -80 ºC. Prior to freezing, an aliquot of each iWAT tissue sample was selected for stroma-vascular fraction (SVF) isolation. Blood samples were also collected, and serum samples were obtained and frozen at -80ºC for biochemical determinations. All experiments were performed according to National Animal Care guidelines, with the approval of the Ethics Committee for Animal Experimentation of the University of Navarra (protocol no. 113–15), and in accordance with the EU Directive 2010/63/EU.

### Treadmill running protocol

The DIOEX group was subjected to a treadmill (LE8710M; Panlab, Barcelona, Spain) training program from 6 months up to 18 months of age as previously described [26]. Prior to the beginning of the treadmill protocol, mice were adapted to treadmill by running two consecutive days for 10 min (first day at 3 m min^−1^, second day at 4.8 m min^−1^). Then, 6 months old DIOEX animals started the treadmill running protocol (3 m min^−1^ for 5 min, increased to 4.8 m min^−1^ for 5 min, and then reaching a maximum of 7.2 m min^−1^ for 20 min; 0% slope) 3 alternate days/week. At 10 months of age, sessions were incremented to 5 days/week for 5 weeks and the protocol was intensified (5 m min^−1^ for 5 min, followed by 8 m min^−1^ for 5 min and 12 m min^−1^ for 20 min; 0% slope). During the next 7 months, sessions were decreased to 3 days/week, and the program was maintained, according to previous studies [99]. During the 12-month training program, untrained DIO mice were left on the treadmill for the same time (30 min, 3 days/week) without running.

### Body composition

Before the sacrifice, whole-animal body composition was measured by magnetic resonance (EchoMRI-100–700; Echo Medical Systems, Houston, TX, USA), as previously described [55].

### Glucose tolerance test

Prior to the sacrifice, a GTT was conducted by administration of intraperitoneal injections of glucose (1.5 g kg^−1^) to overnight fasted (12 h) animals [83]. Tail vein blood was obtained for glucose determinations prior and post injection (at 30, 60, 90 and 120 min). Glucose levels were measured with a standard glucometer (Accu-Check Advantage blood glucose meter, Roche Diagnostic, Basel, Switzerland).

### Biochemical analyses

Fasting serum circulating levels of glucose, total cholesterol, HDL-cholesterol and β-hydroxybutyrate were determined on a Pentra C200 autoanalyzer (HORIBA ABX, Madrid, Spain), following the supplier’s instructions. Serum free fatty acids were assessed using the Non-Esterified Fatty Acids Kit (FUJIFILM Wako Chemicals Europe GmbH, Neuss, Germany). Insulin levels were determined with a commercially available ELISA kit (Mercodia, Uppsala, Sweden), according to the supplier’s guidelines. LDL-cholesterol was calculated using the Friedewald equation [23], and the HOMA-IR index was calculated as described by Matthews et al*.* [52].

### Flow Cytometry in iWAT stroma vascular fraction

iWAT SVF cells were isolated to analyze cell surface markers by flow cytometry. iWAT samples were cut into small pieces and digested with a collagenase buffer (Sigma-Aldrich; St. Louis, MO, USA) during 45 min. Then, blood cells were lysed by adding Ammonium-Chloride-Potassium (ACK) buffer (Gibco, Invitrogen Corporation; Carlsbad, CA, USA). For SVF isolation, cells were centrifuged at 300 g for 5 min and washed 3 times with DMEM-F12 medium (Gibco) containing 10% fetal bovine serum (FBS, Gibco) and 1% penicillin/streptomycin (Invitrogen). The obtained SVF was disaggregated mechanically and filtered in 70 µm cell strainers (Falcon™, #352350, Thermo Fisher Scientific, Waltham, MA, USA). Then, 100 µl of the obtained cells were incubated with the respective antibodies for 15 min at 4 ºC and FcBlock to prevent non-specific binding of Fc receptor. The quantified populations included B cells (CD19^+^), T cells (CD3^+^), granulocytes (Ly6G/Ly6C^+^) and macrophages (F4/80^High/Low^, CD11b^+^). Antibodies were purchased from Biolegend (San Diego, CA, USA), including CD11b FITC (Clone M1/70), CD19-APC-cy7 (Clone 6D5), CD3-PE-cy7 (Clone 17A2) F4/80-PE (Clone BM8) and Ly6G/Ly6C-APC (Clone RB6-8C5). Afterwards, cells were washed with PBS and centrifuged at 300 g (5 min, 4 ºC). After discarding the supernatant, cells were stained with 7-AAD (#A1310, Invitrogen) to assess cell viability (1/100 dilution in PBS, 5 min, room temperature). Flow cytometry was performed in a FACSCantoII device (Becton Dickinson) and analyzed using the FlowJo software (TreeStar).

### Protein expression

iWAT and iBAT samples were homogenized in 200 µl lysis buffer (Pierce ® RIPA Buffer, Thermo Fisher Scientific) with 10 mM ethylenediaminetetraacetic acid and 100 × phosphatase inhibitor cocktail (Halt™, Thermo Fisher Scientific) by sonication (SONOPULS Ultrasonic homogenizer HD 3100, Bandelin, Berlin, Germany) two times for 10 s each. Samples were centrifuged (20,000 g, 15 min, 4 ºC) to collect the supernatant fraction, and protein concentrations were obtained by quantification of the extracts with the BCA protein assay kit (Thermo Fisher Scientific). Protein extracts (40–60 µg) were resolved by electrophoresis on 12% SDS–polyacrylamide electrophoresis gels and electroblotted onto a polyvinylidene difluoride membrane (Amersham™ Hybond™, GE Healthcare Life Science, Freiburg, Germany), which was blocked with 1% bovine serum albumin (BSA) in TBS (Sigma-Aldrich). Then, membranes were incubated overnight (4 ºC) with primary antibodies for uncoupling protein 1 (UCP1, rabbit, #23841 Abcam, Cambridge, UK), hormone-sensitive lipase (HSL, rabbit, #4107, Cell Signaling, Danvers, MA, USA), phosphorylated-HSL (p-HSL Ser563, #4139, Cell Signaling) and β-Actin (rabbit, #SAB5500001, Sigma-Aldrich). Secondary goat anti-rabbit IgG HRP (#1705046, Bio-Rad, Munich, Germany) was used (1 h, room temperature). Thereafter, immunoreactivity was detected with enhanced chemiluminescence (Thermo Fisher Scientific) and quantified by densitometry analysis (Imagen Studio Lite; LI-COR Biosciences, Lincoln, Ne., USA).

### RNA isolation and qRT-PCR

iWAT and iBAT total RNA was extracted with QIAzol lysis reagent® protocol (Qiagen; Venlo, Limburg, The Netherlands) and eluted in Rnase-free DEPC-treated water (Thermo Fisher Scientific). RNA quality and quantity were measured on the Nanodrop Spectrophotometer ND1000 (Thermo Fisher Scientific), and then 2 µg were incubated (30 min, 37 °C) with Dnase I (Thermo Fisher Scientific) and reverse transcribed to cDNA using the High-Capacity cDNA reverse transcription kit (Applied Biosystems, Foster City, CA, USA) following the suppliers’ instructions.

Real-time PCR assays were performed using the Touch Real-Time PCR System (C1000 + CFX384, Bio-Rad, Hercules, CA, USA). Gene expression was analyzed using Taqman Universal Master Mix (Applied Biosystems) methodology for predesigned Taqman Assays-on-Demand, and Power SYBR® Green PCR (Bio-Rad) methodology was used for primers designed with Primer-Blast software (NCBI, MD, USA, https://www.ncbi.nlm.nih.gov/tools/primer-blast/).

For oligonucleotides sequences and references of genes assessed, refer to Supplementary Tables 1 and 2. Genes assayed included: *Fatty acid synthase* (*Fasn), Diacylglycerol O-acyltransferase 1* (*Dgat1), Stearoyl-Coenzyme A desaturase 1 (Scd1), Lipoprotein lipase (Lpl*), *Patatin-like phospholipase domain containing 2* (*Pnpla2*, symbol *Atgl), Lipase, hormone sensitive (Lipe*, symbol *Hsl*), *Carnitine palmitoyltransferase 1a, liver (Cpt1a), Acyl-Coenzyme A oxidase 1, palmitoyl (Acox1), tumor necrosis factor (Tnf), Toll-like receptor 4 (Tlr4), Interleukin 4 (Il4), Interleukin 6 (Il6), Interleukin 10 (Il10), Chemokine (C–C motif) ligand 2 (Ccl2), Integrin alpha X (Itgax*, symbol *Cd11c), mannose receptor, C type 1 (Mrc1,* symbol *Cd206), Adiponectin (Adipoq), Leptin (Lep), Peroxisome proliferative activated receptor, gamma, coactivator 1 α (Ppargc1a,* symbol *Pgc1a), PR domain-containing protein 16 (Prdm16), Uncoupling protein 1 (Ucp1), Transcription factor A, mitochondrial (Tfam), Nuclear respiratory factor 1 (Nrf1), Tumor necrosis factor receptor superfamily, member 9 (Tnfrsf9*, symbol *Cd137), T-box 1 (Tbx1), Transmembrane protein 26 (Tmem26), Fibroblast growth factor 21 (Fgf21), klotho beta (Klb,* symbol *β-Klotho), Fibroblast growth factor receptor 1 (Fgfr1), Early growth response 1 (Egr1), Fibronectin Type III Domain Containing 5 (Fndc5).* Genes’ relative expression was determined by the 2^−ΔΔCt^ method [49] after normalization to *36b4* gene expression.

### Statistical analysis

Statistical analyses were performed using GraphPad Prism 9.0 (Graph Pad software, La Jolla, CA, USA). Comparisons between groups were analyzed with Student’s *t* or Mann–Whitney’s *U* test after testing for normality with Shapiro–Wilk tests. Differences were considered significant at *P* value < 0.05.

## Results

### Body composition and serum metabolic biomarkers of aged, obese exercised mice

Table 1 shows the effects of long-term exercise training on body composition and serum biomarkers of lipid metabolism in 18 months old DIO mice. No differences were observed on food intake between the untrained and trained groups. As observed, the exercised mice (DIOEX group) tended to reduce body weight and fat mass (g), although no statistical differences were reached. No significant changes were either found in lean mass, as previously reported [51] Accordingly, the weights of visceral depots (gonadal, retroperitoneal and mesenteric [26]) and subcutaneous WAT depots were slightly but not significantly reduced, and no changes were observed in iBAT mass. Also, exercise did not promote significant changes in fasting serum levels of triglycerides nor in total, LDL- and HDL-cholesterol, β-hydroxybutyrate, or free fatty acids (Table 1).

**Table 1:** Effects of long-term treadmill exercise on body weight, fat mass, and fat depots weights, as well as on serum lipid profile in 18 months old DIO female mice

	DIO	DIOEX
Body weight (g)	51.19 ± 2.53	48.11 ± 2.41
Body weight gain (g)	25.48 ± 3.15	23.51 ± 1.88
Food intake (g)	2.57 ± 0.05	2.50 ± 0.07
Fat mass (g)	27.69 ± 2.16	25.91 ± 1.92
Interscapular BAT (g)	0.26 ± 0.03	0.24 ± 0.03
Subcutaneous WAT (g)	2.41 ± 0.24	2.21 ± 0.28
Gonadal WAT (g)	3.82 ± 0.40	3.23 ± 0.38
Mesenteric WAT (g)	1.14 ± 0.13	1.19 ± 0.16
Retroperitoneal WAT (g)	0.78 ± 0.14	0.80 ± 0.10
Triglycerides (mg/dl)	66.67 ± 3.94	74.00 ± 7.87
Total chol (mg/dl)	134.78 ± 7.33	137.10 ± 6.11
LDL-cholesterol (mg/dl)	67.48 ± 5.07	64.96 ± 4.79
HDL-cholesterol (mg/dl)	53.96 ± 3.04	57.38 ± 2.41
Free fatty acids (mmol/l)	0.32 ± 0.02	0.28 ± 0.01
β-hydroxybutyrate (mmol/l)	1.32 ± 0.11	1.61 ± 0.23

DIO: diet-induced obese; DIOEX: diet-induced obese + exercise; BAT: brown adipose tissue; WAT: white adipose tissue. Data are mean ± SEM. (n = 6–10)

**Fig. 1:**
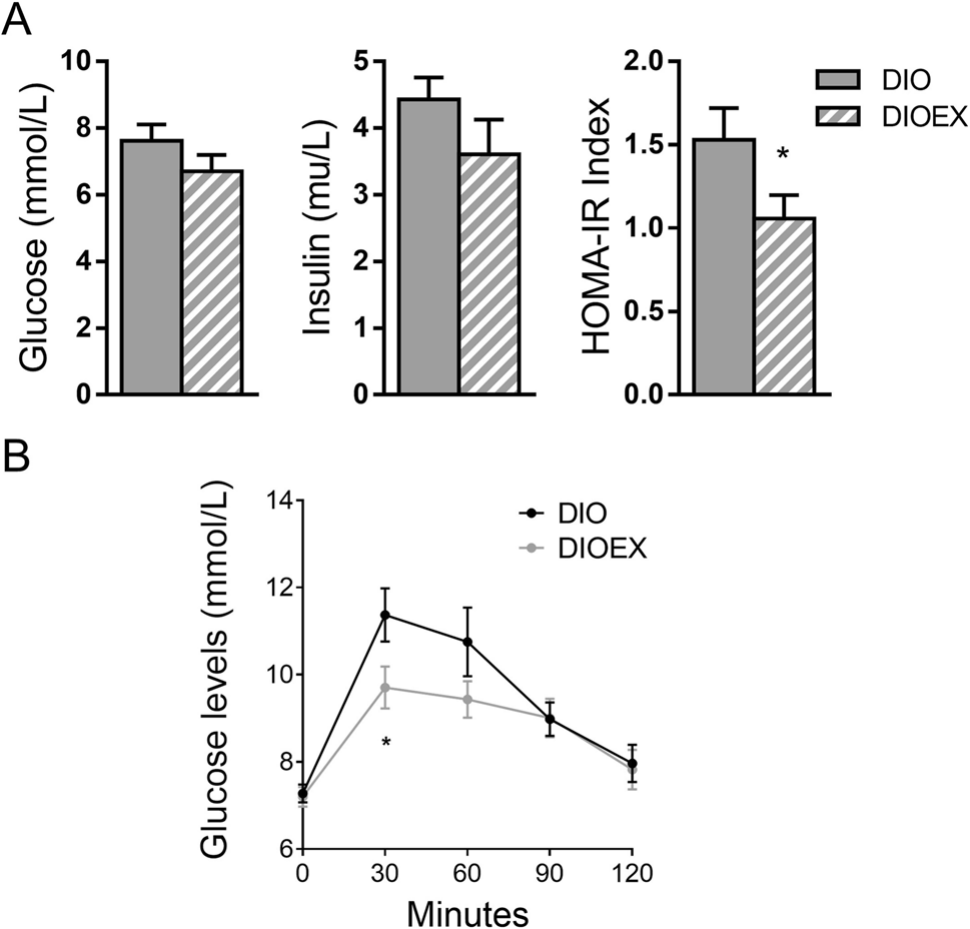
Effects of long-term exercise training on glucose homeostasis and insulin resistance in 18 months old DIO female mice. **A**. Fasting glucose and insulin, as well as the derived HOMA-IR index. **B**. Glucose excursion curves after the glucose tolerance test. Data are mean ± SEM. (n = 6–9). ^*^P < 0.05

Regarding glucose homeostasis, fasting glucose and insulin levels were moderately but not significantly reduced in the aged, trained *vs*. the untrained DIO group (Fig. 1A). However, the DIOEX mice showed a significant reduction in the HOMA-IR, an index for insulin resistance (Fig. 1A), which agrees with the significantly lower peak of glucose (30 min post glucose injection) observed in the GTT excursion curve (Fig. 1B).

### Exercise promotes fatty acid oxidation genes in iWAT, but not in iBAT of aged, obese mice

**Fig. 2:**
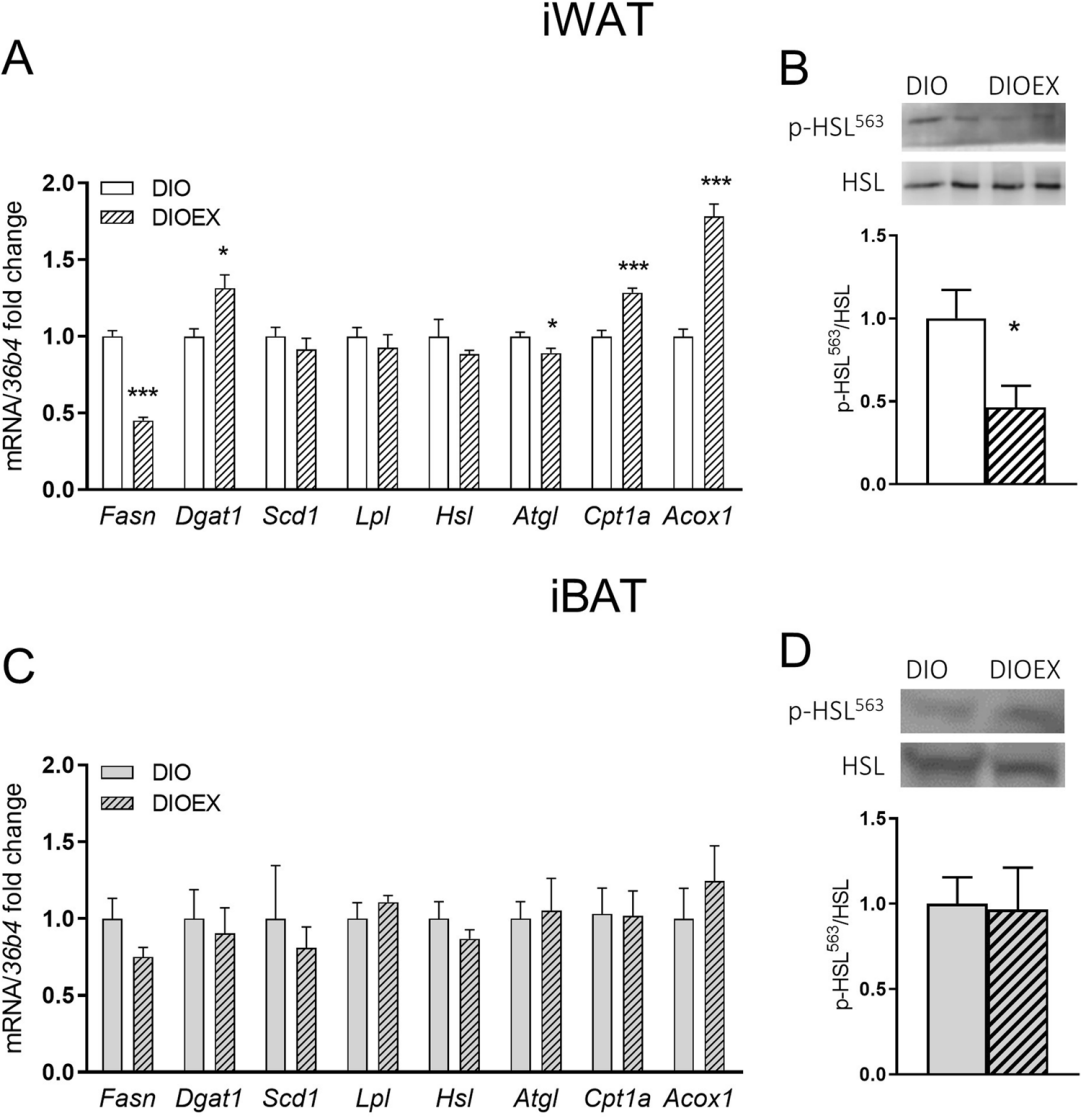
Effects of long-term exercise training on genes controlling fat accumulation and deposition, and on the phosphorylation levels of HSL at Ser^563^ in iWAT (**A, B**) and iBAT (**C, D**) of 18 months old DIO female mice. Data are mean ± SEM. (n = 6–8). ^*^P < 0.05, ^***^P < 0.001

The balance between lipid anabolic (lipogenesis) and catabolic pathways (lipolysis and fatty acid oxidation) is determining adipocyte and adipose tissue size [36]. The regulation of these processes seems to be adipose depot dependent [10, 78]. A comparative study of the effects of exercise on the expression of key lipolytic, lipogenic and fatty acid oxidation genes and proteins was performed between iWAT (Fig. 2A, B) and iBAT (Fig. 2C, D). As observed, iWAT was more responsive than iBAT to the training program. The expression levels of genes or proteins in the DIOEX mice were expressed as fold-change of those of DIO mice, considered as 1, both in iWAT and iBAT (Fig. 2A and 2C). The differential mRNA expression levels between iWAT and iBAT in DIO mice for the different genes evaluated in the manuscript are shown in Supplementary Figure 1.

In iWAT, the effects of long-term exercise on genes promoting fat accumulation in adipocytes were contradictory. Thus, a reduction was observed on *Fasn,* which could suggest a decreased de novo synthesis of fatty acids. However, *Dgat1,* involved in triglycerides esterification, was upregulated, and no changes were observed on *Lpl* in the DIOEX group. The treadmill running program had marginal effects on lipolytic genes, showing no effects on *Hsl* and a minor but significant decrease in *Atgl* (Fig. 2A). Nevertheless, a significant reduction was observed on the phosphorylation levels of HSL at Ser^563^, leading to a decrease in the pHSL^563^/total HSL ratio in iWAT (Fig. 2B), suggesting a reduction on the activation of lipolysis in iWAT of exercised mice. However, fatty acid oxidation genes *Acox1* and *Cpt1a* were markedly increased in the iWAT of the DIOEX group (Fig. 2A). On the contrary, the exercise program did not induce any significant change on the expression levels of any of these genes or in pHSL/HSL ratio in iBAT (Fig. 2C, D).

### Effects of long-term exercise training on local inflammation in iWAT and iBAT

**Fig. 3:**
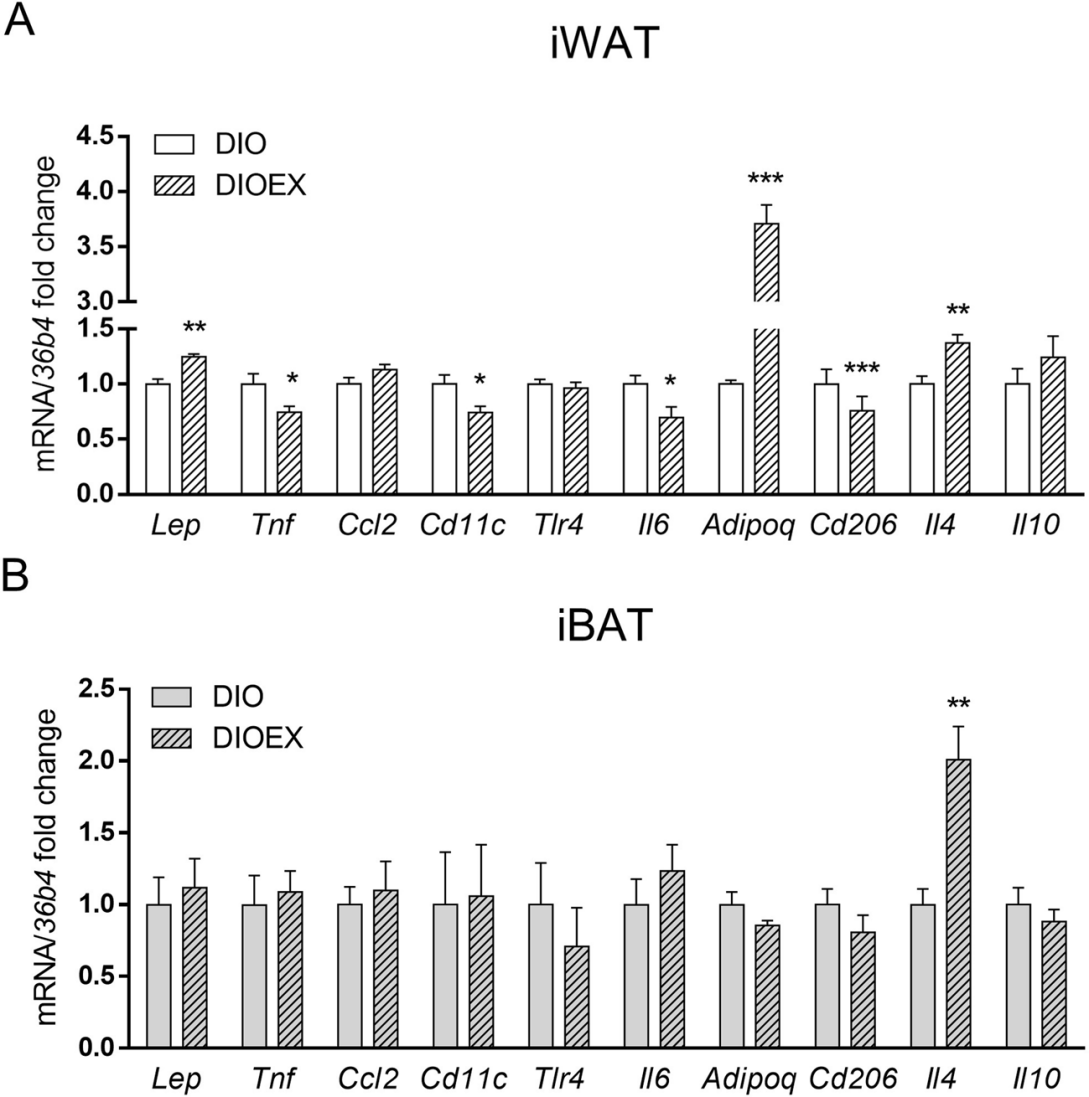
Effects of long-term exercise training on pro and anti-inflammatory genes in iWAT (**A**) and iBAT (**B**) in 18 months old DIO female mice. Data are mean ± SEM. (n = 6–8). ^*^P < 0.05, ^**^P < 0.01, ^***^P < 0.001

To characterize if treadmill training started at late adulthood could attenuate the inflammatory microenvironment associated to obesity and aging in iWAT and iBAT, the gene expression of pro- and antiinflammatory adipocytokines, chemokines and macrophages markers was studied (Fig. 3).

Importantly, several proinflammatory cytokines (*Tnf*, *Il6*) were decreased by exercise in iWAT from trained mice. By contrast, the expression of antiinflammatory adipocytokines (*Adipoq* and *Il4*) was significantly increased (Fig. 3A). Unexpectedly, *leptin* mRNA levels, which are usually positively associated with adiposity and inflammation [60], were moderately but significantly upregulated by exercise training (Fig. 3A), despite iWAT mass tended to be reduced (Table 1). However, other proinflammatory (*Ccl2*, *Tlr4*) and anti-inflammatory (*Il10*) genes showed similar expression levels in both experimental groups (Fig. 3A).

**Fig. 4:**
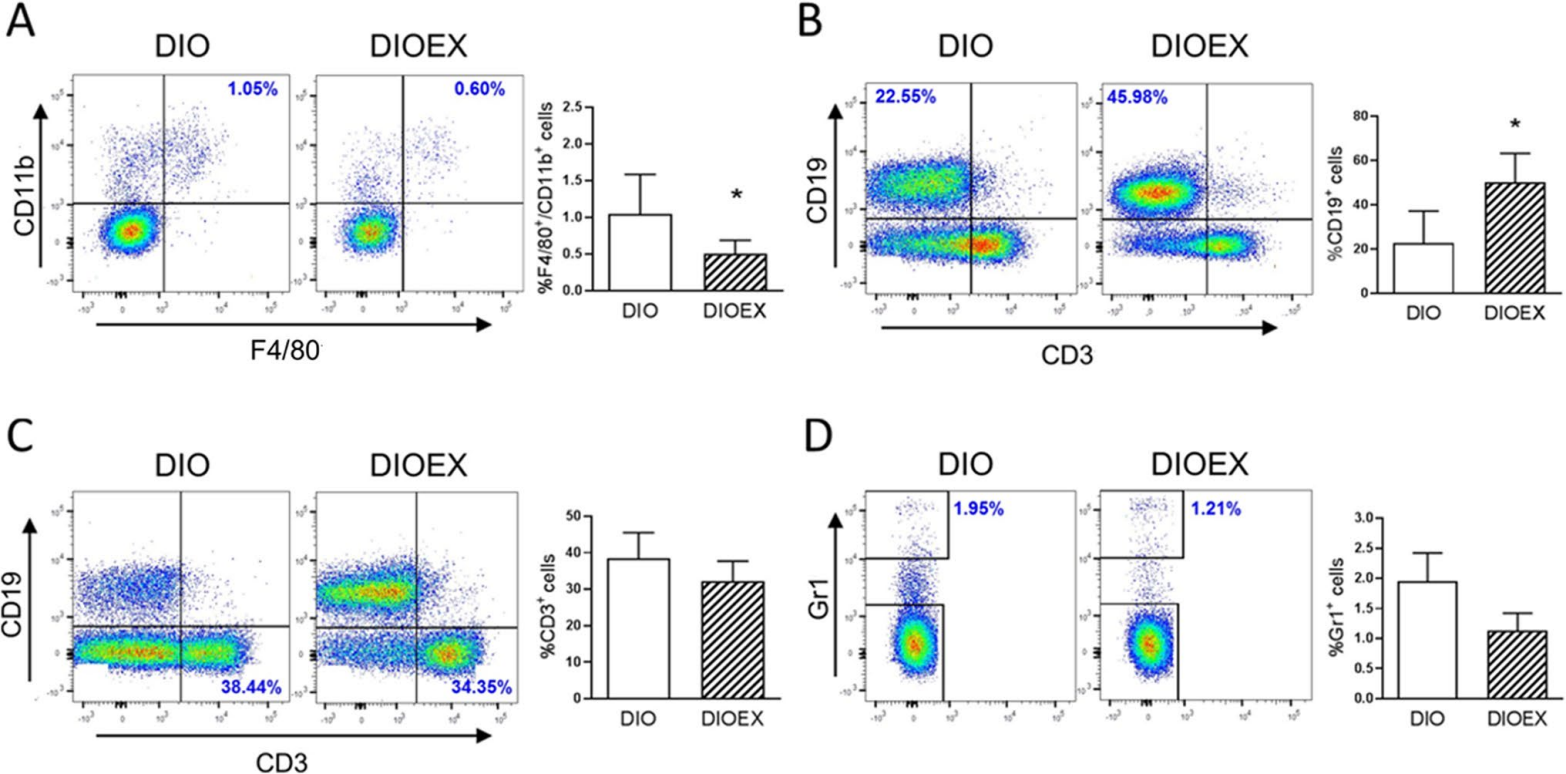
Effects of long-term exercise training on immune cell populations in iWAT. A-D: Analysis of adipose tissue SVF by flow cytometry. Representative dot plots and quantification of **A**. total macrophages (F4/80^+^/CD11b^+^), **B**. B lymphocytes (CD19^+^/CD3^−^), **C**. T lymphocytes (CD19^−^/CD3^+^) and **D**. granulocytes (Gr1^+^) in relation to total immune cells. Data are mean ± SEM. (n = 5–8). ^*^P < 0.05

Furthermore, the levels of markers of M1 proinflammatory macrophages (*Cd11c*) and M2 antiinflammatory macrophages (*Cd206*) were both decreased in DIOEX mice, suggesting a reduced infiltration of macrophages in this fat depot in response to exercise. Indeed, the characterization of the immune cell populations in iWAT by flow cytometry revealed a decrease in total macrophages (F4/80^+^/CD11b^+^) and an increase in B lymphocytes (CD19^+^) in obese exercise-trained mice. However, no changes were observed for T lymphocytes (CD3^+^) or granulocytes (Gr1^+^) in the DIOEX group as compared to the DIO group (Fig. 4).

On the other hand, iBAT depot was also less responsive to the exercise program concerning to inflammation-related genes, as only one of the studied genes revealed significant changes. The results showed an upregulation of the antiinflammatory *Il4* mRNA levels in DIOEX group as compared to untrained DIO mice (Fig. 3B).

### Exercise-induced thermogenic and mitochondrial adaptations in iWAT and iBAT of aged obese mice

**Fig. 5:**
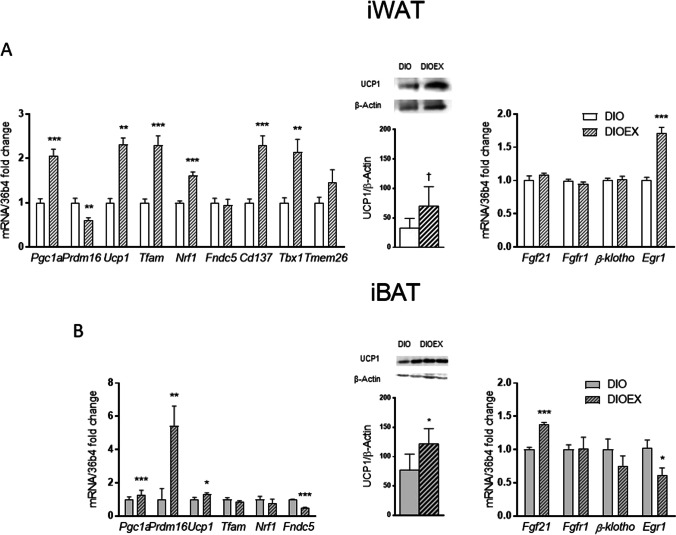
Effects of long-term exercise training on thermogenic function markers in iWAT and iBAT of 18 months old DIO female mice. **A**. mRNA levels of genes involved in mitochondrial biogenesis (*Pgc1a, Tfam, Nrf1*), thermogenic function and adipose tissue browning (*Prdm16, Ucp1, Fndc5*) and beige adipocytes selective genes (*Cd137, Tbx1 and Tmem26*) (Left panel) in iWAT, as well as protein levels of the thermogenic UCP1 (middle panel) in iWAT. The right panel shows mRNA levels of *Fgf21* and signaling pathway genes in iWAT of DIO and DIOEX mice. **B**. iBAT genes involved in mitochondrial biogenesis and thermogenic function (left panel) and UCP1 protein levels (middle panel). The right panel shows mRNA levels of *Fgf21* and signaling pathway genes in iBAT of DIO and DIOEX mice. Data are mean ± SEM. (n = 6–8). ^*^P < 0.05, ^**^P < 0.01, ^***^P < 0.001; ^†^*P* = 0.057

We next aimed to characterize if long-term exercise could induce browning markers in iWAT of obese aged mice, which are known to be reduced by obesity and aging [6, 88]. Our data show that the iWAT of trained DIOEX mice exhibited increased expression of genes related to mitochondrial biogenesis (*Pgc1a*, *Tfam*, *Nrf1*), thermogenic function (*Ucp1*), and beige-specific genes (*Cd137*, *Tbx1*) as compared to the DIO group. By contrast, *Prdm16*, a regulator of the thermogenic program in subcutaneous WAT [5], was moderately decreased in DIOEX *vs*. DIO mice, while the levels of *Fndc5*, which encodes for irisin, were not modified (Fig. 5A, left panel). However, the levels of the thermogenic protein UCP1 tended to be increased (*P* = 0.057) in DIOEX mice (Fig. 5A, middle panel). Moreover, the mRNA levels of *Fgf21, Fgfr1* and *β-klotho* were unaltered, while *Egr1*, a downstream effector of FGF21 signaling, was upregulated in iWAT from DIOEX animals as compared to the DIO group (Fig. 5A, right panel).

Exercise training induced some common and some differential effects on iBAT as compared to those observed on iWAT. Thus, iBAT of DIOEX mice showed increased gene expression of *Pgc1a* and *Prdm16* as well as higher UCP1, both at gene and protein levels, while *Tfam* and *Nrf1* were unaltered, and *Fndc5* levels were significantly downregulated (Fig. 5B, left and middle panels). Moreover, *Fgf21* levels were significantly upregulated in the exercise-trained group, while no changes were observed in its receptors *Fgfr1* and *β-klotho*. However, the levels of *Egr1* were decreased in the DIOEX group as compared to the DIO group (Fig. 5B, right panel).

## Discussion

We here show for the first time how a treadmill running protocol conducted for 12 months in DIO mice induces a differential remodeling in the aged obese iWAT and iBAT, characterized by a more thermogenic and antiinflammatory phenotype.

It is worth mentioning that these actions occurred even in the absence of significant changes either in body weight or in fat mass, as only moderate trends towards a reduction in both parameters were found. Apparently, this contrasts with other studies observing significant body weight and WAT depots decreases, and even BAT mass increases, in response to different types of training in younger animals (6–10 months old) [42, 48, 68, 77, 87, 95]. Few studies have investigated the effects of exercise at older ages. Among them, a study in 23 months old lean mice exercised for the short –term (10 weeks) revealed no significant body weight changes, but a reduction in epididymal WAT compared to their sedentary counterparts [100]. Other studies analyzing the actions of long-term exercise (started at 2 [58] or 12 months of age [54]), have shown body weight and fat mass reductions at 24 and 26 months of age. However, the experimental conditions were different to those of our study, since they were conducted in aged lean mice that followed a voluntary wheel-running training [54, 58]. Only one study has been performed in aged obese animals, by feeding 48 weeks-old mice with a HFD for 16 weeks and starting a 8-weeks treadmill running protocol afterwards. Also in this experimental setting, exercise induced significant reductions in body weight and fat mass [2]. Although our exercise program lasted 12 months, it could not fully counteract the increase in adiposity induced by the HFD diet. However, our HFD feeding began at 2 months of age and was maintained until the end of the experiment for a total of 16 months, which might be the cause of the observed results. Finally, it should be also considered that our study has been performed in female mice and some studies have suggested a sexual dimorphism in response to exercise. In this way, the study of Nigro et al. [57] reported that exercise training did not change body weight, lean mass, or fat mass in females, whereas in males, exercise caused reductions in fat mass and increases in lean mass, with a moderate trend to body weight reduction.

Nonetheless, the exercise training protocol was effective in improving glucose homeostasis, as revealed by the reduction in the HOMA-IR and the lower glucose peak observed for the GTT’s excursion curve. These observations agree with previous studies reporting beneficial effects of exercise on glucose metabolism biomarkers after short and long-term exercise protocols in adult DIO animals [29, 42, 67, 74, 96]. To our knowledge, this is the first time that improvements in glucose metabolism and insulin resistance are reported after exercise training in aged DIO animals [2]. Importantly, these actions occurred in parallel to several exercise-induced adaptations in adipose tissue that were more pronounced in the iWAT than the iBAT depot. In this way, the study of Stanford et al*.* [81] described a crucial role for subcutaneous WAT in mediating the improvements on glucose homeostasis induced by exercise. Thus, transplantation of subcutaneous WAT from exercised animals to sedentary ones, both fed a chow or a HFD, had no effect on the animals’ body weight or composition but improved their glucose homeostasis and insulin resistance [81]. In this way, our current data suggest that, even in the background of obesity and aging, long-term exercise could induce relevant adaptations in iWAT that may have accounted for the beneficial effects on glucose homeostasis and insulin resistance. These iWAT adaptations included an increase in fatty acid oxidation and mitochondrial biogenesis genes, the induction of beige adipocytes markers, and the reduction of inflammation in the exercised 18 months old DIO animals.

An intriguing finding was the upregulation induced by exercise on *Dgat1*, which suggests a promotion of fatty acid esterification and accumulation as triglycerides in iWAT. In the context of both the chronic HFD and the aging-induced subcutaneous WAT loss, the promotion of fat deposition in subcutaneous WAT could be a beneficial adaptation to buffer the fat excess and prevent its ectopic accumulation in other metabolic tissues like the liver, as previously reported in these animals [97]. However, exercise also caused a significant reduction in *Fasn*, a master regulator of de novo lipogenesis that suggests an inhibitory effect of exercise on novel fatty acid formation, which agrees with previous studies in visceral WAT depots of exercised adult DIO animals [15, 67]. On the other hand, the exercise program caused a small downregulation in *Atgl*, and lower phosphorylation of HSL at Ser^563^, which is involved in the activation of this lipase [24]. These data suggest reduced lipolysis in iWAT of exercised mice. However, no significant changes were observed on circulating free fatty acids levels, and therefore we cannot rule out that exercise might be inducing lipolysis in other visceral WAT depots. The exercise-induced effects in hormone-mediated lipolysis are controversial. In one hand, the stimulation of lipolysis by exercise has been demonstrated in some studies in younger DIO animals [3, 15, 48]. However, depot-dependent effects characterized by the stimulation of lipolysis in visceral WAT but not in iWAT have been also described [10]*.* Furthermore, other studies have suggested a decrease in lipolysis revealed by lower circulating non-esterified fatty acids and/or glycerol levels after exercising DIO animals [9, 18, 67]. However, it has also been shown that exercise increases whole body lipolysis and fatty acid oxidation even in the absence of adipose tissue lipolysis [98]. Altogether, these data suggest that the classic role of WAT as an energy fuel supplier, *i.e.* fatty acids due to an enhanced lipolysis during exercise [37], might not make sense in a context of chronic overfeeding.

Concerning fatty acid oxidation, the circulating levels of β-hydroxybutyrate showed a trend to increase, suggesting a stimulation of fatty acid oxidation in exercised DIO mice. In this way, our study showed that long-term exercise significantly increased genes involved in peroxisomal and mitochondrial fatty acid oxidation (*Acox1*, *Cpt1a*) in iWAT, as described by previous studies in visceral and/or iWAT depots of exercised adult DIO rodents [15, 40, 67] and aged DIO mice [2]. A similar upregulation of *Acox1*, and *Cpt1b* was observed in gastrocnemius muscle of DIOEX mice [51]. These group of mice also exhibited increased expression of *Acox1* and *Ppara* in liver [97], further supporting than long-term exercise promotes fatty acid oxidation in obese aged mice. This increment in fatty acid oxidation genes occurred along with significant increases in genes involved in mitochondrial biogenesis (*Pgc1a, Nrf1, Tfam*), in beige adipocytes-specific genes (*Cd137*, *Tbx1*) and in the main thermogenic gene *Ucp1*, suggesting that the long-term moderate exercise program promoted WAT browning in obese aged mice. Although growing evidence has supported the effects of exercise in promoting WAT browning in adult [4, 27, 40, 69, 90] DIO animals, our current data are relevant since they suggest that exercise started in the late adulthood and maintained until the old age can also be effective to prevent the well-established loss of beige adipocytes induced by aging [101], especially in an obesogenic environment.

A moderate increase in the expression of leptin (*Lep*) mRNA was observed in iWAT in response to exercise. This upregulation of leptin occurred in an independent manner of the trends towards a fat mass reduction observed in the DIOEX mice. Although most of the studies have described leptin reductions secondary to fat mass losses after moderate to intense exercise protocols [19, 29], others have found controversial results describing increases in *Lep* mRNA in retroperitoneal fat after endurance training in rats [18]. In this regard, some studies have reported that administration of leptin induces the expression of UCP1 in WAT [13]. Therefore, the upregulation of *Lep* could also contribute to the upregulation of iWAT thermogenic genes in the DIOEX group. However, as a cytokine leptin also regulates immune cells and promotes inflammatory responses [44].

Inflammation of WAT has also been described to impair beige adipogenesis and browning of white adipocytes [88, 89]. Our current data show that long-term exercise was able to counteract the iWAT inflammation that accompanies obesity and aging, as revealed by: i) the lower macrophages content (F4/80^+^/Cd11b^+^) in iWAT SVF; ii) the decrease observed in proinflammatory cytokines (*Tnf*, *Il6)*; and iii) the increase in antiinflammatory adipocytokines *Adiponectin* (*Adipoq)* and *Il4* mRNA levels. Similar antiinflammatory effects have been widely described in the current literature for different types of exercise in the iWAT and visceral WAT depots of adult DIO animals [50, 70, 74, 90]. Concerning the mechanisms by which exercise could promote beige adipocytes markers in iWAT, the observed local increases in adiponectin and IL-4 could be involved, since both have been shown to promote browning of subcutaneous WAT by inducing proliferation of M2 macrophages [35, 63]. However, the reduced macrophage infiltration in the DIOEX group occurred together with a reduction of both M1 proinflammatory (*Cd11c)* and M2 antiinflammatory (*Cd206*) macrophages markers, suggesting that long-term exercise reduced total macrophage content without inducing a polarization of M1 to M2 macrophages. The reduction in total macrophages has been observed in other studies in iWAT and epididymal WAT from adult exercised DIO animals [90, 91, 96]. Only a study reported an increase in CD206 levels in subcutaneous WAT from 6 months old DIO rats exercised for 10 weeks [42], together with a decrease in the M1 marker CD86, suggesting a switch from the M1 to the M2 macrophage phenotype as a consequence of exercise. However, the differences in the animal models of obesity [17], the period of the HFD feeding prior to the beginning of the exercise, and the differences in the training protocol could explain the differential outcomes observed with our current study.

Importantly, the flow cytometry analysis of iWAT SVF also found higher markers of B lymphocytes (CD19^+^) in DIOEX mice. Studies have established that the accumulation of B cells in visceral WAT is one of the earliest responses to HFD, mediating a worsening of glucose tolerance, insulin resistance, and an increased secretion of proinflammatory cytokines that in turn activate both T cells and macrophages to mediate inflammation (reviewed by Srikakulapu and McNamara [79]). However, the role of B lymphocytes in inflammation is, like that of macrophages, subset-dependent (B1-antiinflammatory or B2-proinflammatory), and the different subsets’ abundance in WAT has not been well established yet [79]. Despite these interrelations between macrophages, B and T lymphocytes, we could not find any significant changes in T cells (CD3^+^) in SVF after exercise in the DIOEX group. Significant decreases in T cells have been found in previous studies, but using other surface markers (CD8^+^, CD4^+^) and in younger exercised DIO animals [39, 91]. Hence, it seems that the long-term exercise program promoted an antiinflammatory local environment in iWAT of obese aged mice mainly by stimulating an antiinflammatory signaling profile and reducing macrophages markers, while the role of B lymphocytes remains to be elucidated. Several studies have shown an inflammation-driven stimulation of lipolysis in obese WAT of both mice and humans [30, 34]. Therefore, the exercise-induced improvement in the inflammatory state could contribute to the reduction in lipolysis observed in iWAT of DIO mice.

On the other hand, our study also reveals that iBAT is less sensitive than iWAT to the training protocol. Indeed, no changes were observed on genes related to lipid metabolism nor in most of those associated with iBAT inflammatory status. Among the genes that were similarly regulated by exercise in iWAT and iBAT, *Pgc1a* and *Ucp1* were included, suggesting an activation of the thermogenic response in both fat depots. Another common response between iWAT and iBAT was the upregulation of the anti-inflammatory cytokine *Il4* in exercised animals. As discussed above, IL-4 signaling has been described to play a major role in development of functional beige fat, whereby the genetic loss of IL-4/13 signaling alters the cold-induced beige fat biogenesis, while IL-4 administration increases beige fat mass and its thermogenic capacity in obese mice [63]. Therefore, the upregulation observed on *Il4* could also be underlying the browning properties of long-term exercise both in iWAT and iBAT.

Our data also revealed a differential upregulation of *Prdm16* between iWAT and iBAT. Indeed, a marked upregulation was observed only in iBAT of the exercised DIO animals. Actually, PRDM16 levels are typically higher in brown than in white adipocytes, since it is responsible for developing and maintaining the identity of brown fat cells in adulthood [31, 75]. Therefore, the induction of *Prdm16* by exercise could help to promote BAT recruitment and prevent the loss of brown fat characteristics that occurs during obesity and aging, as recently reported by our group [20].

A differential expression was also observed in irisin encoding gene *Fndc5*, which was not changed in iWAT, but was decreased in iBAT in response to exercise. There is strong evidence for irisin mediating browning of iWAT when released from skeletal muscle after exercise in young mice [8]. Importantly, we have recently reported an increase in *Fndc5* gene expression in the gastrocnemius muscle of these exercised aged obese mice [51]. However, the regulation of *Fndc5*/irisin in WAT after exercise is conflictive [11, 66, 85], and the different duration and the type of exercise make difficult the comparison of effects between studies. Interestingly, other studies have suggested that the Fndc5 increase in response to exercise training seems to occur in rodents fed standard but not high-fat diet [69, 93]. Regarding the effects of exercise on *Fndc5*/irisin in iBAT, a study has reported an increase after 6 weeks of volunteer-wheel running in iBAT of adult male Balb-c mice, which contrast with our observation of reduced *Fndc5* expression after long-term treadmill exercise in obese old mice [85].

The regulation of *Fgf21* and its signaling pathways in response to chronic exercise was also different between iWAT and iBAT. The iBAT of the aged DIOEX group showed an increase in *Fgf21*, as previously reported in younger obese exercised mice [94]. The stimulation of FGF21 in BAT has been related to an increase in its thermogenic activity [33]. Therefore, the observed upregulation of *Fgf21* could be related to the higher UCP1 expression observed in the trained mice’ iBAT. In the different WAT depots, FGF21 has been also identified as a browning inducer [21, 64]. In this sense, exercise has been shown to restore FGF21 signaling in WAT of young (4–5 months old) DIO mice [94]. Moreover, the beneficial effects of exercise on WAT metabolism have been related with an increased FGF21 sensitivity, mediated by an increment in the levels of the FGF21 receptor components FGFR1 and β-klotho [25]. However, our data show that long-term exercise did not have any effect on iWAT’s *Fgf21* mRNA levels nor on the expression of *Fgfr1* or *β-klotho*, suggesting that the stimulation of *Fgf21* signaling did not mediate the browning of iWAT observed in DIOEX mice. Nevertheless, the increased iWAT expression of *Erg1*, a canonical downstream effector of FGF21 pathway, seems to argue against this possibility. Such stimulation could suggest a greater sensitivity to FGF21 after exercise in obese mice, as proposed by Geng et al*.* [25]*.* In fact, in this study the authors found that *Egr1* was upregulated in BAT and WAT of exercised, but not of sedentary obese mice injected with intraperitoneal FGF21 [25]*.* However, to our knowledge there are no studies in the literature analyzing the changes in *Egr1* in response to exercise per se in BAT. In this regard, we observed an adipose depot-dependent regulation of *Egr1* by the long-term exercise program. Hence, in contrast to what was observed in iWAT, *Egr1* was downregulated in iBAT of exercised mice. Nonetheless, it is worth mentioning that the role of *Egr1* in adipose tissue has not been fully established yet. In this respect, the observation that *Egr1* deficiency promotes WAT browning specifically in the subcutaneous depot, while BAT oxidative metabolism remains unchanged [7], highlights the relevance to carry out future studies to uncover the function of *Egr1* on the different adipose depots.

There are limitations to this work to be acknowledged. First, the use of female mice limits our discovery to one sex. The biology of sex difference has already identified differences also in the adaptations of adipose tissue to exercise [22, 57]. On the other hand, our study was conducted at subneutral temperatures (below 30 °C), and several studies have suggested that the effects of exercise on whole body, muscle and adipose tissue (mainly brown fat) are influenced by temperature [53, 65]. Future studies should consider housing temperatures in the experimental design of studies investigating mice exercise physiology. Finally, a comparative study about the effects of exercise on visceral WAT *vs.* iWAT would have been of interest considering that visceral WAT accumulation is harmful for metabolic health and it is increased during aging [43, 61].

## Conclusions

Long-term exercise promotes a beneficial remodeling of iWAT in aged obese mice, characterized by the modulation of pro and antiinflammatory signaling genes towards an antiinflammatory profile, the reduction of macrophage infiltration, and the upregulation of genes involved in fatty acid oxidation, mitochondrial biogenesis, and adipose tissue beiging. Although the exercise program also induces an upregulation of thermogenic markers in iBAT, this depot seems to be less responsive to exercise than iWAT. The adaptations induced in iWAT and iBAT could contribute to the improvements in glucose tolerance and insulin resistance observed in aged exercise-trained mice. These data suggest that moderate exercise started in late adulthood and maintained until old age could ameliorate the deleterious effects of obesity and aging on white, beige and brown fat, thus helping to prevent metabolic disturbances even in an obesogenic environment.

## Supplementary Information

Below is the link to the electronic supplementary material.

Supplementary file1 (DOCX 142 KB)
