# Supplementary material for: Differential remodeling of subcutaneous white and interscapular brown adipose tissue by long-term exercise training in aged obese female mice
Source: J Physiol Biochem. 2023 May 19;79(2):451–65. doi: 10.1007/s13105-023-00964-2 (PMC10300187; doi:10.1007/s13105-023-00964-2)
Supplement: Supplementary file 1 — Supplementary file1 (DOCX 142 KB) [file 13105_2023_964_MOESM1_ESM.docx]

**Differential remodeling of subcutaneous white and interscapular brown adipose tissue by long-term exercise training in aged obese female mice**

*Journal of Physiology and Biochemistry*

Elisa Félix-Soriano^1^, Neira Sáinz^1^, Eva Gil-Iturbe^1^, Rosa Castilla-Madrigal^1^, Jon Celay^2,3^, Marta Fernández-Galilea^1^, Álvaro Pejenaute^1,5^, M. Pilar Lostao^1,4,5^, José A Martínez-Climent^2,3,4^, María J Moreno-Aliaga^1,4,5,*^

^1^ University of Navarra; Center for Nutrition Research and Department of Nutrition, Food Science and Physiology; School of Pharmacy and Nutrition. Pamplona, Spain.

^2^ Division of Hemato-Oncology, Center for Applied Medical Research CIMA, University of Navarra, Pamplona, Spain.

^3^ CIBERONC, Instituto de Salud Carlos III (ISCIII), Madrid, Spain.

^4^ IdiSNA, Navarra Institute for Health Research, Pamplona, Spain.

^5^ CIBEROBN, Instituto de Salud Carlos III (ISCIII), Spain.

^*^Correspondence: [mjmoreno@unav.es](mailto:mjmoreno@unav.es); Tel.: +34 948 425 600, Ext. 806558.

**Supplementary Table 1.** NCBI primer blast-designed oligonucleotides used for qRT-PCR analyses.

| **Gene symbol** | **Forward sequence** | **Reverse sequence** |
| --- | --- | --- |
| *Acox1* | 5’-CTATGGGATCAGCCAGAAAG-3’ | 5’-AGTCAAAGGCATCCACCAA-3’ |
| *Adipoq* | 5’-AAGGGAGAGAAAGGAGATGC-3’ | 5’-TACACATAAGCGGCTTCTCC-3’ |
| *Ccl2* | 5’-AGCACCAGCCAACTCTCACT-3’ | 5’-TCATTGGGATCATCTTGCTG-3’ |
| *Cd206* | 5’-CAAGGAAGGTTGGCATTTGT-3’ | 5’-CCTTTCAGTCCTTTGCAAGC-3’ |
| *Cpt1a* | 5’-CACCAACGGGCTCATCTTCTA-3’ | 5’-CAAAATGACCTAGCCTTCTATCGAA-3’ |
| *Dgat1* | 5’-GAGGCCTCTCTGCCCCTATG-3’ | 5’-GCCCCTGGACAACACAGACT-3’ |
| *Fasn* | 5’-GCTGCGGAAACTTCAGGAAAT-3’ | 5’-AGAGACGTGTCACTCCTGGACTT-3’ |
| *Egr1* | 5’-GTCCTTTTCTGACATCGCTCTGA-3’ | 5’-CGAGTCGTTTGGCTGGGATA-3’ |
| *Fgf21* | 5’-CCTCTAGGTTTCTTTGCCAACAG-3’ | 5’-AAGCTGCAGGCCTCAGGAT-3’ |
| *Fgfr1* | 5’-TACAAGGTTCGCTATGCCAC-3’ | 5’-TGCGGAGATCGTTCCACGAC-3’ |
| *Fndc5* | 5’-GGTGCTGATCATTGTTGTGG-3’ | 5’-CGCTCTTGGTTTTCTCCTTG-3’ |
| *Il10* | 5’-AAGGCAGTGGAGCAGGTGAA-3’ | 5’-CCAGCAGACTCAATACACAC-3’ |
| *Il4* | 5’-ACAGGAGAAGGGACGCCAT-3’ | 5’-GAAGCCCTACAGACGAGCTCA-3’ |
| *Il6* | 5’-GAGGATACCACTCCCAACAGACC-3’ | 5’-AAGTGCATCATCGTTGTTCATACA-3’ |
| *Itgax (Cd11c)* | 5’-ACGTCAGTACAAGGAGATGTTGGA-3’ | 5’-ATCCTATTGCAGAATGCTTCTTTACC-3’ |
| *Klb (β-klotho)* | 5’-ACGACCCGACGAGGGCTGTT-3’ | 5’-GGAGGAGACCGTAAACTCGGGCTTA-3’ |
| *Lipe (Hsl)* | 5’-CTGCTTCTCCCTCTCGTCTG-3’ | 5’-CAAAATGGTCCTCTGCCTCT-3’ |
| *Lpl* | 5’-GCCAAGAGAAGCAGCAAGAT-3’ | 5’-CCATCCTCAGTCCCAGAAAA-3’ |
| *Nrf1* | 5’-GCTCACTTCCTCCGGTCCTTTG-3’ | 5’-GACAAGATCATCAACCTGCCTGTAG-3’ |
| *Ppargc1a (Pgc1a)* | 5’-CTAGCCATGGATGGCCTATTT-3’ | 5’-GTCTCGACACGGAGAGTTAAAG-3’ |
| *Prdm16* | 5’-CAGCCATACAGGTGCAAGTA-3’ | 5’-GAACGGCTTCTCTTTGTTGTG-3’ |
| *Tlr4* | 5’-TGGTTGCAGAAAATGCAGG-3’ | 5’-AGGAACTACCTCTATGCAGGG-3’ |
| *Tnf* | 5’-CATCTTCTCAAAATTCGAGTGACAA-3’ | 5’-TGGGAGTAGACAAGGTACAACCC-3’ |
| *Ucp1* | 5’-ACTGCCACACCTCCAGTCATT-3’ | 5’-CTTTGCCTCACTCAGGATTGG-3’ |

**Supplementary Table 2.** TaqMan Assays-on-Demand oligonucleotides used for qRT-PCR analyses.

| **Gene symbol** | **Catalog number** |
| --- | --- |
| *Lep* | Mm00434759_m1 |
| *Pnpla2 (Atgl)* | Mm00503040_m1 |
| *Scd1* | Mm00772290_m1 |
| *Tbx1* | Mm00448949_m1 |
| *Tfam* | Mm00447485_m1 |
| *Tmem26* | Mm01173641_m1 |
| *Tnfrsf9 (Cd137)* | Mm00441899_m1 |

**Supplementary Fig. 1.** Differential expression of genes controlling fat accumulation and deposition (**A**), pro and anti-inflammatory genes (**B**) and thermogenic function markers (**C**) in iWAT and iBAT of 18 months old DIO female mice. Data are mean ± SEM. (n=6-9). ^***^*P*<0.001 ^**^*P*<0.01, ^*^*P*<0.05.
